# Overexpression of Aurora-A in primary cells interferes with S-phase entry by diminishing Cyclin D1 dependent activities

**DOI:** 10.1186/1476-4598-10-28

**Published:** 2011-03-16

**Authors:** Florian Jantscher, Christine Pirker, Christoph-Erik Mayer, Walter Berger, Hedwig Sutterluety

**Affiliations:** 1 Institute of Cancer Research, Comprehensive Cancer Center, Medical University of Vienna, Borschkegasse 8a, A-1090 Vienna, Austria; 2 Helmholtz Zentrum München (German Research Center for Environmental Health), Marchioninistrasse 25, D-81377 Munich, Germany

## Abstract

**Background:**

Aurora-A is a bona-fide oncogene whose expression is associated with genomic instability and malignant transformation. In several types of cancer, gene amplification and/or increased protein levels of Aurora-A are a common feature.

**Results:**

In this report, we describe that inhibition of cell proliferation is the main effect observed after transient overexpression of Aurora-A in primary human cells. In addition to the known cell cycle block at the G2/M transition, Aurora-A overexpressing cells fail to overcome the restriction point at the G1/S transition due to diminished RB phosphorylation caused by reduced Cyclin D1 expression. Consequently, overexpression of Cyclin D1 protein is able to override the Aurora-A mediated G1 block. The Aurora-A mediated cell cycle arrest in G2 is not influenced by Cyclin D1 and as a consequence cells accumulate in G2. Upon deactivation of p53 part of the cells evade this premitotic arrest to become aneuploid.

**Conclusion:**

Our studies describe that an increase of Aurora-A expression levels on its own has a tumor suppressing function, but in combination with the appropriate altered intracellular setting it might exert its oncogenic potential. The presented data indicate that deactivation of the tumor suppressor RB is one of the requirements for overriding a cell cycle checkpoint triggered by increased Aurora-A levels.

## Background

The family of the Aurora/Ipl1p kinases is evolutionally conserved. These serine/threonin kinases fulfill important functions in the control and regulation of the centrosome cycle, spindle assembly, chromosome condensation, microtubule-kinetochore attachment, the spindle-assembly checkpoint, cytokinesis, as well as entry into and exit from mitosis. In mammals, the Aurora kinase family comprises three members designated Aurora-A, -B, and -C [1-3].

One member of this family, Aurora-A (AURKA), also known as serine/threonine kinase 15 (STK15), aurora2 or aurora related kinase (hARK1), is decisively involved in centrosome duplication, separation, as well as maturation [4]. Its functions are required to ensure progression through mitosis [5-7], complete cytokinesis [8] and genomic integrity [4,9].

The gene encoding Aurora-A maps to chromosome 20q13.2, a region that is frequently amplified in human cancers including colorectal [10], breast [4], pancreatic [11] and bladder cancer [12]. Consequently, mRNA and protein levels of Aurora-A are also increased in those types of carcinoma. In addition, overexpression of Aurora-A has been found in a variety of human tumors and cancer cell lines, independent of gene amplification [4,13] (reviewed in [14]). In accordance, ectopic Aurora-A expression transforms immortalized NIH 3T3 cells in tissue culture [4,10].

Although accumulating evidence emphasizes an oncogenic role of overexpressed Aurora-A in carcinogenesis, several reports describe that in primary mouse models Aurora-A overexpression often fails to induce cancer, even in p53 deficient animals. In mammary glands, periodic Aurora-A overexpression has been shown to cause mitotic abnormalities and massive apoptosis. Hyperplasia of the surviving cells was observed, however no malignant tumors developed [15]. Another study reports that Aurora-A expression even failed to induce hyperplasia [16]. It was furthermore observed that increased Aurora-A expression in liver caused premitotic arrest during liver regeneration [17]. Corroborating knock-out studies revealed that Aurora-A may also act as a haploinsufficient tumor suppressor. Whereas Aurora-A null mice died early during embryonic development, Aurora-A heterozygosity resulted in a significantly increased tumor incidence [18].

In this report, we studied the effect of Aurora-A overexpression in primary human cells and show that high levels of Aurora-A inhibit cell proliferation at both G1/S and G2/M transition.

## Results

### Overexpression of Aurora-A inhibits cell proliferation in primary human cells

**Figure 1:**
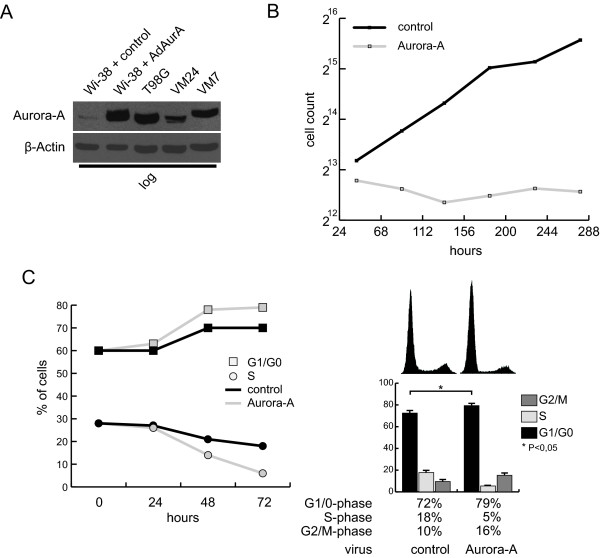
**Influence of Aurora-A overexpression on cell cycle**. (A) Wi-38 cells were infected with adenoviruses expressing either Aurora-A or control (lacZ) proteins. Overexpressed protein levels were compared to T98G (glioblastoma), VM24, and VM7 (melanoma) cell lines. (B) Wi-38 cells overexpressing Aurora-A were tested in a growth curve experiment against cells infected with a control virus. The results of two independent experiments are depicted. (C) Logarithmically growing Wi-38 cells were infected with the viruses expressing the indicated proteins and DNA content was measured 24, 48 and 72 hours after infection. The time curve shows the percentage of cells in G1 and S-phase from a representative experiment. The histograms and the respective cell cycle analysis of the 72 hours time point is depicted. Bars illustrate means and standard deviation of four independent experiments. Statistical analysis was done using an unpaired, two-sided t-test. * P < 0.05.

Our initial interest was directed towards the influence of ectopic Aurora-A overexpression on cell proliferation and cell cycle profile of primary human lung fibroblasts (Wi-38 cells). To this end, Aurora-A expression in logarithmically growing Wi-38 cells was elevated by using an adenoviral vector to reach protein levels observed in tumor cell lines with gains of chromosome 20q, including the gene locus of AURKA (Figure 1A) [19]. By performing a growth curve experiment 24 hours post-infection, the influence of Aurora-A overexpression on cell proliferation was determined. While cells infected with the control virus expressing lacZ increased their number six-fold within 9 days, Aurora-A expression inhibited proliferation efficiently. Within 10 days the number of cells remained unchanged (Figure 1B). These data suggest that Aurora-A overexpression actually inhibits cell proliferation of primary human cells.

To investigate at which phases of the cell cycle Aurora-A overexpression inhibits proliferation, PI-staining of logarithmically growing Wi-38 cells was performed 24, 48 and 72 hours after infection of the cells with lacZ and Aurora-A expressing viruses (Figure 1C). Flow cytometric analysis of the DNA content revealed that as a consequence of Aurora-A overexpression the amount of cells in S-phase successively declines (Figure 1C, left panel). In addition to an increase of cells with doubled DNA content (16% compared to 10%), we detected an obvious and significant accumulation of cells in G1 phase in response to Aurora-A overexpression after 72 hours (79% compared to 72%) (Figure 1C, right panel). These data indicate that Aurora-A might not only be involved in a checkpoint at the G2/M transition, but can also interfere with cell cycle progression in G1 phase.

### S-phase entry is inhibited by Aurora-A expression in quiescent cells

**Figure 2:**
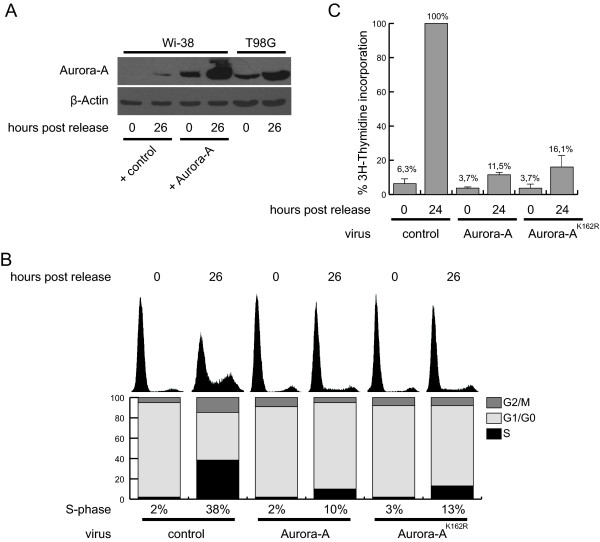
**G1 cell cycle arrest mediated by Aurora-A wildtype and K162R**. (A) Serum starved Wi-38 cells were infected with adenoviruses expressing either Aurora-A or control (lacZ) proteins. Cell were induced by serum addition and protein levels were compared to those of equally treated T98G cells 26 hours post induction (B) Arrested Wi-38 cells were infected with adenoviruses expressing the proteins indicated. 48 hours after infection serum was added and PI-stainings were collected. A representative of three experiments is shown. (C) Cells were treated as in (B) and a ^3^H-thymidine incorporation assay was performed. Bars depict means and SD of three independent experiments.

To confirm the observed inhibitory effect of Aurora-A in G1 phase, Wi-38 cells were serum starved for 24 hours before infection with the Aurora-A adenovirus. Again, the adenoviral titer necessary to reach Aurora-A expression levels similar to those in a serum starved tumor cell line (T98G) was determined (Figure 2A).

Aurora-A expression had no influence on the amount of cells in quiescence. 48 hours after infection serum starved Wi-38 cells were released from G0 phase by addition of growth medium containing 20% serum. 26 hours after serum addition, more than 50% of the cells exited quiescence in control cells (38% in S- and 15% in G2-phase), while in Aurora-A overexpressing cells only about 10% of cells entered S-phase (Figure 2B). Accordingly, ^3^H-thymidine incorporation assay revealed that upon serum stimulation incorporation of thymidine was only slightly increased (3-fold) when cells express Aurora-A, whereas control cells have about 15-fold higher incorporation rates than their arrested counterparts (Figure 2C). These data verify the results from the PI stainings and demonstrate that overexpression of Aurora-A in quiescent cells prevents DNA replication by causing either defects in G1-phase exit or S-phase entry.

### Kinase activity is dispensable for the inhibitory function of Aurora-A prior to S-phase entry

To investigate if the observed interference with induction of DNA replication is connected to the kinase activity of overexpressed Aurora-A protein, quiescent Wi-38 cells were infected with adenoviruses expressing Aurora-A mutated at amino acid 162 to Arg (AurA^K162R^). This mutant had previously been described as kinase deficient [8]. Similar to the wildtype (wt) protein, expression of AurA^K162R ^repressed S-phase entry of quiescent cells (Figure 2B). Corresponding to the DNA content analysis, initiation of DNA replication was inhibited by AurA^K162R ^as measured by thymidine incorporation (Figure 2C). These data indicate that the kinase activity is not required for the blockage of cells in G1 phase in response to expression of Aurora-A protein.

### The G0/G1 arrest is not caused by the interplay of Aurora-A with p53, RASSF1 or RasGAP

**Figure 3:**
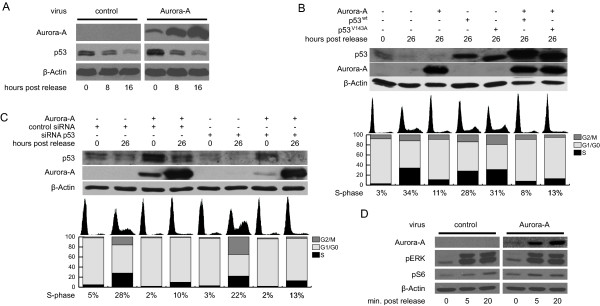
**Importance of known Aurora-A interactions for cell cycle inhibition**. (A) Wi-38 cells were arrested in G0 by serum deprivation and infected with adenoviruses expressing Aurora-A or control virus expressing lacZ and analyzed by immunoblotting with the antibodies indicated. (B) Quiescent cells were infected with adenoviruses expressing the indicated proteins and analyzed by immunoblotting and PI staining at time points 0 and 26 hours after serum addition. (C) Serum arrested cells expressing Aurora-A or lacZ were transfected with the indicated siRNA 24 hours prior to serum addition. At the indicated time points cells were harvested and analyzed as in (B). (D) Serum deprived cells infected with control or Aurora-A expressing viruses were harvested at the indicated times and analyzed by immunoblotting with antibodies recognizing phosphorylation of ERK1/2 and ribosomal protein S6, respectively. For all illustrations, representatives of at least two independent experiments are shown.

Previous studies have shown that Aurora-A directly binds to p53. Although the reported effects of this interaction are phosphorylation-dependent inhibition of p53 function [20] and stability [21], we next examined if the triggering of an G0/G1 arrest by overexpression of Aurora-A in serum-deprived cells is influenced by p53 levels or functions. Immunoblotting demonstrated that p53 levels in arrested, Aurora-A overexpressing Wi-38 cells were increased compared to control cells (Figure 3A). However, since after serum addition p53 expression declined more rapidly in Aurora-A overexpressing cells, after 16 hours the p53 protein levels were comparable to control cells (Figure 3A). Nonetheless it is possible that elevated p53 levels in arrested cells could be responsible for the Aurora-A-mediated obstruction of cell cycle progression in G0/G1. Adenoviral expression of p53^V143A ^protein, which was described to be dominant negative with respect to the transcriptional activities [22], was utilized to evaluate the involvement of p53 in the observed G1-arrest. As control, cells expressing p53^wt ^protein were analyzed. As anticipated, overexpression of p53^wt ^decelerated cell cycle progression of control cells. In combination with p53^wt ^the Aurora-A triggered inhibition of G0/G1 exit was yet effective. Even though we observed an accelerated cell cycle progression of control cells when p53 functions were inhibited, p53^V143A ^failed to abrogate the Aurora-A-mediated cell cycle stop (Figure 3B). In addition, p53 expression levels were reduced by transfecting siRNA. Although the p53 protein levels were efficiently decreased in cells treated with a p53-specific siRNA (lanes 7 and 8 in Figure 3C) as compared to cells transfected with a control siRNA (lanes 3 and 4 in Figure 3C), cells predominantly arrested in G0/G1-phase when Aurora-A was overexpressed (Figure 3C). These data suggest that p53 is not responsible for the inhibitory function of Aurora-A prior to transition from G1- to S-phase.

Two other established interaction partners of Aurora-A protein, RASSF1 [23] and RasGAP proteins [24], are known to associate with and modulate Ras activity and are therefore possibly involved in the inhibition of DNA replication by excessive amounts of Aurora-A. We hence analyzed if Aurora-A influences Ras-mediated signaling pathways by monitoring phosphorylation of ERK1/2 (extracellular signal-regulated kinase) for MAPK (mitogen-activated protein kinase) pathway and phosphorylation of ribosomal protein S6 for the PI3K (phospho-inosityl-3-kinase) pathway. To this end, quiescent Aurora-A expressing and control cells were induced by addition of serum. After 5 and 20 minutes the intensity of the pERK signaling was comparable in both control and Aurora-A virus-treated cells. Similar intensities of phosphorylated ribosomal protein S6 levels were observed after 20 minutes. After 5 minutes phosphorylation of S6 was even enhanced in Aurora-A expressing cells (Figure 3D). These data suggest that expression of Aurora-A does not interfere with activation of Ras-mediated signaling indicating that the interaction with RASSF1 and RasGAP cannot explain the inhibitory role of Aurora-A in cell cycle progression.

### Aurora-A overexpression in quiescent cells negatively influences Cyclin D1-mediated activities

**Figure 4:**
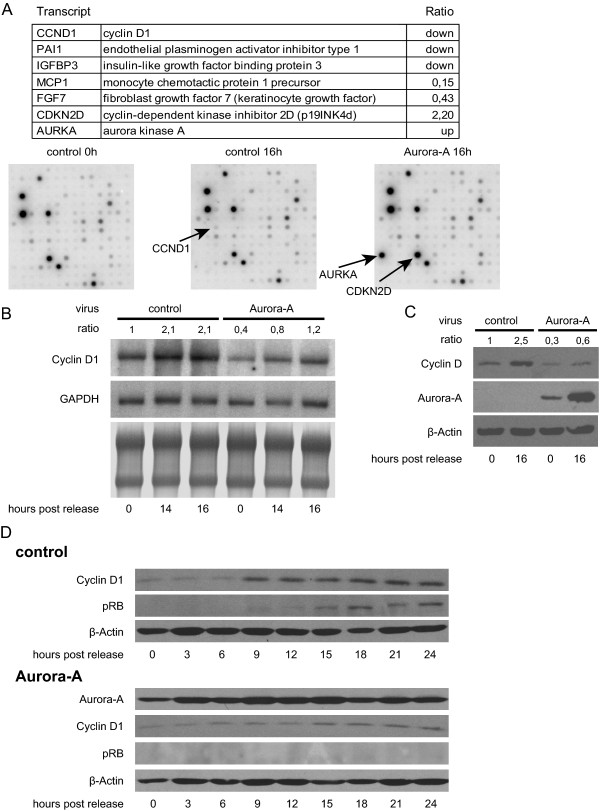
**Identification of modulated gene expression caused by Aurora-A expression during G0/G1 progression**. 24 hours after serum removal, arrested Wi-38 cells were infected with adenoviruses expressing either Aurora-A or lacZ (control). Serum was added 48 hours after infection and cells were harvested at the times indicated. Total RNA was extracted and Clonetech human Atlas 1.2 cDNA expression array analyses were performed. (A) A list of the genes, which fail to respond to serum addition in Aurora-A overexpressing cells is presented in the upper panel. Ratio given indicates the difference in mRNA expression compared to controls. The lower panel highlights the region of the array containing the probes for Cyclin D1 and p19^INK4d^. (B) Verification of the array data was done using Northern blot analysis with a Cyclin D1 probe spanning the first 350 nucleotides of the Cyclin D1 coding sequence and a probe for GAPDH from nucleotides 192 to 549 for normalization. One of 3 Northern blots from independent experiments is shown. The expression level ratios of Cyclin D1 were calculated after densitometric analysis using ImageQuant software (Molecular Dynamics, Sunnyvale, CA) and normalized to GAPDH. Serum-deprived control cells were set to 1. (C) Immunoblot of equally treated cells was performed and assayed for Cyclin D1 protein expression. (D) At the indicated times, immunoblotting of serum released cells expressing either lacZ (control) or Aurora-A was performed with the antibodies indicated.

To elucidate which regulatory pathways prior to S-phase entry were affected by Aurora-A overexpression, we analyzed the expression of molecules involved in cell cycle regulation. RNA from lacZ and Aurora-A expressing cells in G0 and late G1 phase (16 hours after serum induction) was extracted to perform an expression analysis using Human BD Atlas cDNA expression array 1.2.1. In response to serum addition, 65 of the genes assayed by the expression array changed their mRNA levels more than 2-fold in control treated cells. Only 6 of these genes showed a 2-fold difference in expression between Aurora-A expressing and control cells indicating that Aurora-A expression interferes with their response to serum addition. 5 out of those 6 genes were upregulated in response to serum in control cells while their levels in cells expressing Aurora-A were comparable to those in quiescent cells. These genes include Cyclin D1, the fibroblast growth factor-7 (FGF7/KGF/HBGF-7), the monocyte chemotactic protein 1 precursor (MCP1, CCL-2), the insulin-like growth factor binding protein 3 (IGFBP3), and plasminogen activator inhibitor Type 1 (PAI1). p19^INK4 d ^(CDKN2D), a known inhibitor of the Cyclin D/CDK4/6 complexes, was reduced in response to serum addition in control cells, while its levels remained high in Aurora-A expressing cells (Figure 4A). Since we did not observe an influence of Aurora-A expression on MAPK and PI3K pathways (compare Figure 3), we decided to focus on Cyclin D1 involving pathways. Cyclin D1 (CCND1) is known to activate CDK4 and CDK6, a key event in mediating G1/S transition [25]. To confirm the differences detected in the expression arrays, synchronized lacZ and Aurora-A overexpressing Wi-38 cells were analyzed by Northern blot analysis. As expected, in control Wi-38 cells Cyclin D1 levels were increased when quiescent cells were released into the cell cycle. In cells overexpressing Aurora-A, Cyclin D1 mRNA levels were reduced in absence and presence of serum. Although addition of serum resulted in an about 3-fold increase of Cyclin D1 expression the yielded mRNA amounts failed to significantly exceed the levels of serum starved control cells (Figure 4B). Corresponding to the mRNA analysis, Cyclin D1 protein expression in Aurora-A expressing cells was less pronounced and increased only twofold (Figures 4B, 4C). To test whether this observation is exclusive for Wi-38 cells, normal adult skin fibroblasts Hs 545 SK were tested and showed a similar decrease in Cyclin D1 levels upon Aurora-A overexpression (data not shown). These data indicate that expression of Aurora-A might result in repression of Cyclin D1/CDK4/6-mediated kinase activity. Therefore we analyzed the influence of Aurora-A expression on phosphorylation of Serine 795 in the retinoblastoma gene product (RB), a key Cyclin D1/CDK4/6 substrate site [26]. While phosphorylation of RB was observed 18 hours after serum induction in control cells, Aurora-A infected cells exhibited no detectable RB-phosphorylation (Figure 4D). We therefore conclude that a negative influence of Aurora-A expression on Cyclin D1 levels may contribute to diminished RB-phosphorylation.

### Expression of Cyclin D1 overrides the Aurora-A mediated block in G1

**Figure 5:**
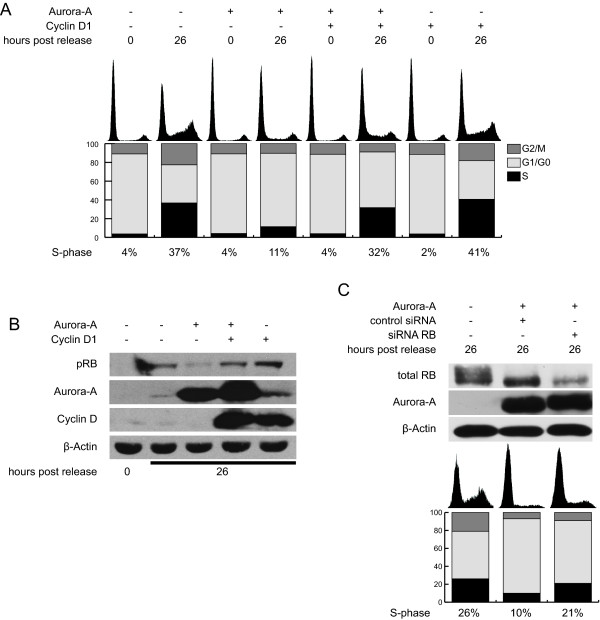
**Influence of Cyclin D1 co-expression along with Aurora-A on G1/S arrest**. G0 arrested Wi-38 cells were infected with the viruses expressing the indicated proteins 24 hours after serum depletion. 48 hours after infection, serum was added. (A) At the indicated time points, PI-stainings were performed. (B) In parallel, cell extracts were analyzed by immunoblotting with the indicated antibodies. A representative experiment of at least three experiments is shown. (C) Serum arrested cells expressing Aurora-A or lacZ were transfected with the control or Rb specific siRNA. 26 hours after serum release, cells were analyzed by immunoblotting and PI-stainings.

To test the significance of the observed reduction of Cyclin D1 activity for the inhibitory role of Aurora-A on cell cycle progression after exit of G0 phase, quiescent Wi-38 cells were infected with viruses expressing lacZ, Aurora-A, Cyclin D1 or the combination of Aurora-A and Cyclin D1. On its own, Cyclin D1 expression had no influence on cell cycle progression of serum induced Wi-38 cells. In contrast, if Aurora-A overexpressing cells were co-infected with viruses coding for Cyclin D1, 40 to 50% of the cells were able to enter the cell cycle in response to serum addition (Figure 5A). Additionally, immunoblot-analysis revealed that Cyclin D1 expression in cells overexpressing Aurora-A resulted in restoration of RB-phosphorylation (Figure 5B). These data demonstrate that Cyclin D1 expression is sufficient to abrogate the G1 phase checkpoint induced by Aurora-A. To assess if restoration of RB phosphorylation is predominantly necessary to overcome the Aurora-A mediated G1-block, we reduced the RB levels using siRNA (Figure 5C). Downregulation of RB increased the proportion of cells able to transit the G1/S boundary, even though the override of the Aurora-A-mediated G1-arrest was less efficient as compared to Cyclin D1 overexpression. This difference may be due to other Cyclin D1 functions or the only partial knock-down of RB protein (see Figure 5C). However, hypophosphorylation of RB is one determinant hindering cell cycle progression in the presence of excessive Aurora-A.

### Cyclin D1 has no effect on the G2/M arrest in response to Aurora-A overexpression

**Figure 6:**
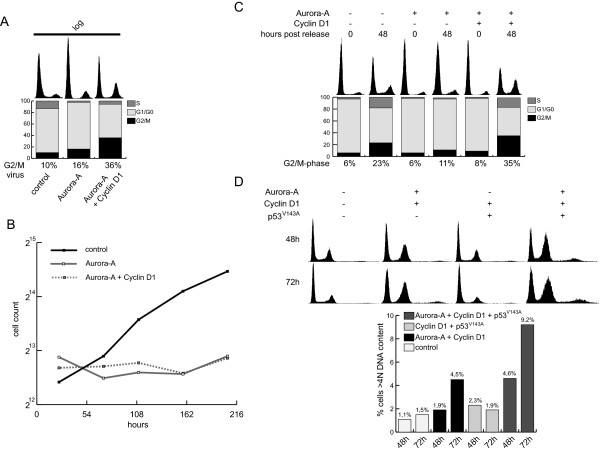
**Long term effects of Aurora-A and Cyclin D1 co-expression**. (A) Logarithmically growing Wi-38 cells were infected with the adenoviruses indicated. Three days after infection, cells were analyzed by PI-staining. One of two data sets is presented. (B) A growth curve experiment was performed with logarithmically growing Wi-38 infected with the viruses indicated. Data of two independent experiments are shown. (C) Serum starved Wi-38 cells were infected with adenoviruses expressing control (lacZ), Aurora-A, Cyclin D1 or a combination of these proteins. Cells were induced by serum addition and analyzed by PI-staining after 48 hours. A representative of three experiments is depicted. (D) Wi-38 cells were treated as in (C), but infected with the indicated combinations of Aurora-A, Cyclin D1 and p53^V143A ^expressing adenoviruses. After 48 and 72 hours, cells were analyzed by PI-staining. Bars depict the percentage of cells with a DNA content >4N. A representative of two experiments is shown.

We subsequently investigated the effect of Cyclin D1 expression on the G2/M arrest in response to Aurora-A overexpression. Therefore, logarithmically growing Wi-38 cells were infected with either Aurora-A expressing viruses alone or with a combination of Aurora-A and Cyclin D1. This Cyclin D1 co-expression with Aurora-A resulted in accumulation of cells in G2/M (~ 35% instead of ~ 15%) after 3 days (Figure 6A). Growth curve analysis revealed that Cyclin D1 fails to override the inhibition of cell proliferation induced by elevated Aurora-A levels, indicating that the G2/M block is independent of Cyclin D1 activity (Figure 6B). To verify this observation, cell cycle progression of cells expressing Aurora-A and/or Cyclin D1 was additionally measured 48 hours after serum addition (Figure 6C). At this time point the majority of the control treated cells have executed mitosis and the G1 peak is again elevated. The cells overexpressing Aurora-A remain blocked in G1. Only the 10 to 15% of cells which are able to enter the cell cycle progress to G2 (compare Figure 2). If Cyclin D1 is co-expressed with Aurora-A, the cells able to evade the G1 block accumulate in G2/M phase indicating that Cyclin D1 expression overrides the Aurora-A mediated G1-, but not the G2-block (Figure 6C).

According to the literature, the mechanisms responsible for the G2/M block are unknown, but it is documented that lack of p53 activity facilitates the escape of the premitotic Aurora-A block [8]. Therefore we tested if expression of dominant negative p53 is able to override the block triggered by concomitant Aurora-A and Cyclin D1 expression in serum released cells. In line with the observations of Meraldi et al. [8], abrogated p53 function resulted in a remarkable increase of cells with a more than 4N DNA content as measured 48 hours and 72 hours after serum addition (Figure 6D). Taken together these results indicate that deactivation of RB and p53 controlled checkpoints are required for the role Aurora-A plays as inducer of aneuploidy.

## Discussion

Aneuploidy is a common characteristic of cancer cells and is considered as an important tumor promoting force. Numerical and structural alterations of chromosomes are often the consequences of failures in a surveillance mechanism called the mitotic checkpoint. In many tumors Aurora-A overexpression is a frequent alteration associated with aneuploidy and chromosomal instability. Thus, Aurora-A has been regarded as an oncogene.

In this study we present data indicating that in primary cells, Aurora-A overexpression in first instance decelerates or blocks cell proliferation. This observation is in line with earlier publications showing that Aurora-A overexpression causes an arrest of cell cycle progression at the G2/M transition [8,17].

In contrast to these earlier reports, we observed that increased levels of Aurora-A protein not only exhibit an inhibitory function during G2/M phase, but additionally interfere with progression through G1 phase. Since in normal cells Aurora-A functions are primarily associated with processes important for regulating G2/M transition, a role of Aurora-A prior to S-phase entry is interesting. Only few publications have hinted that there may be non-mitotic functions of Aurora-A. Its interaction with a histone deacetylase complex induces cilia disassembly [27] and it is part of an aPKC-Aurora-A-NDEL1 pathway playing a role in neuron differentiation [28]. Additionally, a growth inhibitory role of Aurora-A was reported in Drosophila, where Aurora-A inhibits self renewal of neuroblasts [29,30].

Interestingly, the enzymatic activity of Aurora-A seems to be dispensable for its cell cycle suppressing functions. As it has been reported for the G2/M arrest [8], increased levels of the catalytically inactive Aurora-A^K162R ^are also able to activate regulative mechanisms to halt cell cycle progression during G0/G1, inferring that the tumor suppressive roles of the Aurora-A protein are not dependent on its kinase activity. Since the same mutant fails to function in transformation of immortalized rodent cells [10], it is likely that the oncogenic functions of this Aurora family member may be assigned to enhanced kinase activity. Although the elucidated molecular mechanisms involving Aurora-A are connected to its kinase activity, there are data indicating that Aurora-A could exert kinase independent functions [31]. While in case of Aurora-A inactivation by antibody injection cells showed defects in completing cytokinesis [32], inhibition of Aurora-A with a kinase inhibitor did not affect cytokinesis in the analyzed cell lines [33], arguing for the existence of a kinase-independent mode of action.

We observed that the majority of quiescent Aurora-A overexpressing cells fail to exit G0/G1 phase. Concomitantly, Cyclin D1 levels are diminished and RB-phosphorylation does not take place. A fundamental involvement of RB in the G0/G1 block induced by Aurora-A expression can also explain why ectopic Aurora-A expression during liver regeneration only caused a premitotic arrest, but failed to arrest cells prior to S-phase entry [17]. Deletion of RB has been shown to have no impact on hepatic proliferation, while the role of RB in cell cycle control is well established in most other cell models [34].

Since the changes in Cyclin D1 protein and mRNA expression levels are comparable, we can exclude that Aurora-A interactions with components of the protein degradation machinery are responsible for the reduced Cyclin D1 levels. In subsequent studies the exact mechanisms of how Aurora-A expression decreases Cyclin D1 mRNA levels have to be elucidated.

Although Cyclin D1 co-expression can largely override the Aurora-A mediated inhibition of S-phase entry, we cannot exclude that additional defects in other pathways hinder cell cycle progression. Beside the changed expression of Cyclin D1 and p19^INK4d^, four other molecules usually induced by serum addition show a more than twofold change of their expression upon increased Aurora-A levels. The gene products of these candidates are secreted to the cellular environment. Whereas MCP-1 and FGF 7 are usually involved in paracrine signaling pathways [35,36], PAI-1 and IGFBP3 were reported to modulate autocrine signaling cascades [37,38] and may therefore also affect the cell cycle exit of quiescent Wi-38 cells. Yet although activation of MAPK and PI3K pathways was not inhibited by Aurora-A overexpression, it is still possible that the reduced activation of these proteins can influence processes other than RB phosphorylation and thereby explain why Cyclin D1 cannot completely abrogate the inhibitory effect of Aurora-A in G1. Nonetheless, even though re-expression of Cyclin D1 overrides a G1 arrest induced by Aurora-A, restoration of Cyclin D1 activity is not sufficient to overcome arrest at G2/M and subsequently the antiproliferative effect elicited by Aurora-A.

Similar to the G1 arrest, the mechanisms responsible for the inhibition of G2/M transition by excessive Aurora-A levels have not yet been elucidated. However, it was reported that p53 functions are involved in maintenance of this arrest [8], although p53 null cells respond with a premitotic arrest to Aurora-A overexpression [17]. Cells escaping this block undergo tetraploidization [8]. Accordingly, we report that p53 functions are dispensable for the G0/G1 arrest induced by Aurora-A, but expression of dominant negative p53 facilitated the escape of Aurora-A/CyclinD1 co-expressing cells from the premitotic arrest, resulting in the formation of an aneuploid subpopulation.

## Conclusion

It is a common phenomenon that an opportune accumulation of defects can make a protein gain tumor-promoting properties. This can be observed in the case of the Ras oncogenes, which primarily induce senescence [39], but are a strong tumor-promoting force in an appropriately altered intracellular setting. We conclude that an increase of Aurora-A expression levels on its own results in repression of proliferation, most likely by inducing cell cycle checkpoints, but in combination with other alterations, which include deactivation of the tumor suppressor RB, might exert its oncogenic potential.

## Materials and methods

### Cell culture, growth curves and siRNA

Embryonic lung fibroblasts (Wi-38), adult human skin fibroblasts Hs 545 SK (CRL-7318) and the glioblastoma cell line T98G were obtained from ATCC and cultured using DMEM growth medium containing 10% fetal bovine serum (FBS) and supplemented with penicillin (100 U/ml), streptomycin (100 μg/ml) and pyruvate. Melanoma cell lines VM7 and VM24 were established from primary tumor samples and cultured as described [40]. Synchronization of cells in certain phases of the cell cycle was achieved by serum starvation as described in ref. [41]. Growth curves were performed as described elsewhere [42].

For siRNAs transfections 10^5 ^cells were plated in 6-well dishes and serum starved for 24 hours before cells were infected with adenovirus. 48 hours post-infection, siRNAs were transfected using oligofectamine according to the manufacturer's instructions (Invitrogen, Carlsbad, CA). The sequences used were: sip53 5'-GCAUGAACCGGAGGCCCAUTT-3' and siRb 5'-GAUACCAGAUCAUGUCAGATT-3'.

### Adenovirus preparation

Using the vectors kindly provided by Dr. Erich A. Nigg (Max-Planck-Institut für Biochemie, Martinsried), the coding sequences of wild type Aurora-A and the kinase deficient mutant K162R were subcloned into the pADlox recombination vector via BamH1 and Bgl2 sites. The coding sequences of Cyclin D1 and p53 were amplified from human cDNA using the primers 5'-TCTGGGATCCATGGAACACCAGCTCCTGTGCTGC-3' (Cyclin D1 fw), 5'-GTATGAATTCTCAGATGTCCACGTCCCGCACGTC-3' (Cyclin D1 rev), 5'-GAATGGATCCATGGAGGAGCCGCAGTCAGATCC-3' (p53 fw) and 5'-TCTAGAATTCTCAGTCTGAGTCAGGCCCTTCTGTC-3' (p53 rev) and cloned into the pADlox vector via introduced BamH1 and EcoR1 sites. The p53 mutant V143A was prepared by site directed mutagenesis. The constructs were verified by restriction digestion and sequencing. Recombinant viruses were produced as described in ref. [43] and designated AdAurA and AdAurA^K162R^, Adp53^wt^, Adp53^V143A ^and AdCycD1. A virus expressing lacZ was used as a control [42]. All infections were performed at a multiplicity of infection (MOI) of 50 if not indicated otherwise.

### Propidium iodide based DNA content analysis (PI-staining)

Cells were trypsinized and collected by centrifugation. Cell pellets were washed with PBS and fixed in ice cold 70% ethanol. Cells were again centrifuged, washed twice and resuspended in 300 μl PBS and subsequently stained by addition of Propidium iodide (50 μg/ml) and RNase A (500 μg/ml). Measurements were performed on a BD FACScalibur and analyzed with MODFIT software.

### Thymidine incorporation assay

Serum starved cells were infected with recombinant adenovirus and subsequently released into the cell cycle by addition of 20% serum. 17 hours after induction, the growth medium was replaced with serum free medium and to each well [^3^H]-thymidine (1.25 μCi/ml) was added. After incubation for 1 hour, medium was aspirated and cells were harvested in 500 μl lysis buffer (0.2% SDS, 20 mM EDTA). After precipitation of the DNA by addition of 500 μl of 20% TCA, DNA was spotted on fiberglass filters which were then submerged in scintillation liquid. Detection was performed on a Packard 2200TR scintillation counter.

### Protein extraction from cell lines

Protein extracts of cell lines were obtained by adding whole cell extract buffer (20 mM HEPES pH 7.6, 0.4 M NaCl, 1 mM EDTA, 5 mM NaF, 25% Glycerol, 0.1% NP-40) supplemented with 25 μl/ml complete protease inhibitor mix and 2.5 μl/ml sodium orthovanadate to frozen cell pellets. The viscous solution was passed several times through a syringe fitted with a 27G needle. After centrifugation at 13.000 rpm for 15 minutes, the supernatant containing the extracted protein was collected.

### Immunoblotting

Western blot was performed as described elsewhere [41]. Primary antibodies against Aurora-A, pERK, pS6, and pRB were purchased from Cell Signaling (Danvers, MA). p53 and Cyclin D1 antibodies were purchased from Santa Cruz Biotechnology (Santa Cruz, CA) and a ß-Actin antibody was obtained from Novus Biologicals (Cambridge, UK).

### Northern Blot and expression arrays

Total RNA was prepared as described [44]. RNA analyses by Northern blotting were performed as described before [45]. BD Atlas cDNA Expression Array analyses were performed with total RNA according to the manufacturer's protocol (BD Biosciences Clontech, Franklin Lakes, NJ).

## Abbreviations

AURKA-Aurora-A: AurA^K162R ^- Aurora-A mutated at Arginine 162; CDK: cyclin-dependent kinase; ERK: extracellular signal-regulated kinase; MAPK: mitogen-activated protein kinase; PI3K: phospho-inosityl-3-kinase; PI: propidium iodide; p53^V143A^: p53 mutated at Valin 143; RB: retinoblastoma gene product; wt: wildtype.

## Competing interests

The authors declare that they have no competing interests.

## Authors' contributions

FJ performed most of the experiments, participated in designing the study, analyzing the data and drafting the manuscript, CP and CM were involved in cloning and constructing adenoviruses, WB participated in design of the study, HS conceived of the study, and participated in its design and helped to analyse the data and draft the manuscript.

All authors read and approved the final manuscript.

## References

[B1] Adams RR Carmena M Earnshaw WC Chromosomal passengers and the (aurora) ABCs of mitosis Trends Cell Biol 2001 11 49 54 10.1016/S0962-8924(00)01880-8 11166196

[B2] Andrews PD Knatko E Moore WJ Swedlow JR Mitotic mechanics: the auroras come into view Curr Opin Cell Biol 2003 15 672 83 10.1016/j.ceb.2003.10.013 14644191

[B3] Crane R Gadea B Littlepage L Wu H Ruderman JV Aurora A, meiosis and mitosis Biol Cell 2004 96 215 29 10.1016/j.biolcel.2003.09.008 15182704

[B4] Zhou H Kuang J Zhong L Kuo WL Gray JW Sahin A Tumour amplified kinase STK15/BTAK induces centrosome amplification, aneuploidy and transformation Nat Genet 1998 20 189 93 10.1038/2496 9771714

[B5] Hirota T Kunitoku N Sasayama T Marumoto T Zhang D Nitta M Aurora-A and an interacting activator, the LIM protein Ajuba, are required for mitotic commitment in human cells Cell 2003 114 585 98 10.1016/S0092-8674(03)00642-1 13678582

[B6] Dutertre S Cazales M Quaranta M Froment C Trabut V Dozier C Phosphorylation of CDC25B by Aurora-A at the centrosome contributes to the G2-M transition J Cell Sci 2004 117 2523 31 10.1242/jcs.01108 15128871

[B7] Dutertre S Prigent C Aurora-A overexpression leads to override of the microtubule-kinetochore attachment checkpoint Mol Interv 2003 3 127 30 10.1124/mi.3.3.127 14993419

[B8] Meraldi P Honda R Nigg EA Aurora-A overexpression reveals tetraploidization as a major route to centrosome amplification in p53-/- cells Embo J 2002 21 483 92 10.1093/emboj/21.4.483 11847097 PMC125866

[B9] Miyoshi Y Iwao K Egawa C Noguchi S Association of centrosomal kinase STK15/BTAK mRNA expression with chromosomal instability in human breast cancers Int J Cancer 2001 92 370 3 10.1002/ijc.1200 11291073

[B10] Bischoff JR Anderson L Zhu Y Mossie K Ng L Souza B A homologue of Drosophila aurora kinase is oncogenic and amplified in human colorectal cancers Embo J 1998 17 3052 65 10.1093/emboj/17.11.3052 9606188 PMC1170645

[B11] Li D Zhu J Firozi PF Abbruzzese JL Evans DB Cleary K Overexpression of oncogenic STK15/BTAK/Aurora A kinase in human pancreatic cancer Clin Cancer Res 2003 9 991 7 12631597

[B12] Sen S Zhou H Zhang RD Yoon DS Vakar-Lopez F Ito S Amplification/overexpression of a mitotic kinase gene in human bladder cancer J Natl Cancer Inst 2002 94 1320 9 12208897 10.1093/jnci/94.17.1320

[B13] Sakakura C Hagiwara A Yasuoka R Fujita Y Nakanishi M Masuda K Tumour-amplified kinase BTAK is amplified and overexpressed in gastric cancers with possible involvement in aneuploid formation Br J Cancer 2001 84 824 31 10.1054/bjoc.2000.1684 11259099 PMC2363814

[B14] Keen N Taylor S Aurora-kinase inhibitors as anticancer agents Nat Rev Cancer 2004 4 927 36 10.1038/nrc1502 15573114

[B15] Zhang D Hirota T Marumoto T Shimizu M Kunitoku N Sasayama T Cre-loxP-controlled periodic Aurora-A overexpression induces mitotic abnormalities and hyperplasia in mammary glands of mouse models Oncogene 2004 23 8720 30 10.1038/sj.onc.1208153 15480417

[B16] Fukuda T Mishina Y Walker MP DiAugustine RP Conditional transgenic system for mouse aurora a kinase: degradation by the ubiquitin proteasome pathway controls the level of the transgenic protein Mol Cell Biol 2005 25 5270 81 10.1128/MCB.25.12.5270-5281.2005 15923640 PMC1140609

[B17] Li CC Chu HY Yang CW Chou CK Tsai TF Aurora-A overexpression in mouse liver causes p53-dependent premitotic arrest during liver regeneration Mol Cancer Res 2009 7 678 88 10.1158/1541-7786.MCR-08-0483 19435814

[B18] Lu LY Wood JL Ye L Minter-Dykhouse K Saunders TL Yu X Aurora A is essential for early embryonic development and tumor suppression J Biol Chem 2008 283 31785 90 10.1074/jbc.M805880200 18801727 PMC2581543

[B19] Pirker C Lotsch D Spiegl-Kreinecker S Jantscher F Sutterluty H Micksche M Response of experimental malignant melanoma models to the pan-Aurora kinase inhibitor VE-465 Exp Dermatol 19 1040 7 10.1111/j.1600-0625.2010.01182.x 21087322

[B20] Liu Q Kaneko S Yang L Feldman RI Nicosia SV Chen J Aurora-A abrogation of p53 DNA binding and transactivation activity by phosphorylation of serine 215 J Biol Chem 2004 279 52175 82 10.1074/jbc.M406802200 15469940

[B21] Katayama H Sasai K Kawai H Yuan ZM Bondaruk J Suzuki F Phosphorylation by aurora kinase A induces Mdm2-mediated destabilization and inhibition of p53 Nat Genet 2004 36 55 62 10.1038/ng1279 14702041

[B22] Willis A Jung EJ Wakefield T Chen X Mutant p53 exerts a dominant negative effect by preventing wild-type p53 from binding to the promoter of its target genes Oncogene 2004 23 2330 8 10.1038/sj.onc.1207396 14743206

[B23] Rong R Jiang LY Sheikh MS Huang Y Mitotic kinase Aurora-A phosphorylates RASSF1A and modulates RASSF1A-mediated microtubule interaction and M-phase cell cycle regulation Oncogene 2007 26 7700 8 10.1038/sj.onc.1210575 17563743

[B24] Gigoux V L'Hoste S Raynaud F Camonis J Garbay C Identification of Aurora kinases as RasGAP Src homology 3 domain-binding proteins J Biol Chem 2002 277 23742 6 10.1074/jbc.C200121200 11976319

[B25] Sherr CJ Roberts JM Living with or without cyclins and cyclin-dependent kinases Genes Dev 2004 18 2699 711 10.1101/gad.1256504 15545627

[B26] Connell-Crowley L Harper JW Goodrich DW Cyclin D1/Cdk4 regulates retinoblastoma protein-mediated cell cycle arrest by site-specific phosphorylation Mol Biol Cell 1997 8 287 301 9190208 10.1091/mbc.8.2.287 PMC276080

[B27] Pugacheva EN Jablonski SA Hartman TR Henske EP Golemis EA HEF1-dependent Aurora A activation induces disassembly of the primary cilium Cell 2007 129 1351 63 10.1016/j.cell.2007.04.035 17604723 PMC2504417

[B28] Mori D Yamada M Mimori-Kiyosue Y Shirai Y Suzuki A Ohno S An essential role of the aPKC-Aurora A-NDEL1 pathway in neurite elongation by modulation of microtubule dynamics Nat Cell Biol 2009 11 1057 68 10.1038/ncb1919 19668197

[B29] Lee CY Andersen RO Cabernard C Manning L Tran KD Lanskey MJ Drosophila Aurora-A kinase inhibits neuroblast self-renewal by regulating aPKC/Numb cortical polarity and spindle orientation Genes Dev 2006 20 3464 74 10.1101/gad.1489406 17182871 PMC1698452

[B30] Wang H Somers GW Bashirullah A Heberlein U Yu F Chia W Aurora-A acts as a tumor suppressor and regulates self-renewal of Drosophila neuroblasts Genes Dev 2006 20 3453 63 10.1101/gad.1487506 17182870 PMC1698451

[B31] Lens SM Voest EE Medema RH Shared and separate functions of polo-like kinases and aurora kinases in cancer Nat Rev Cancer 10 825 41 10.1038/nrc2964 21102634

[B32] Marumoto T Honda S Hara T Nitta M Hirota T Kohmura E Aurora-A kinase maintains the fidelity of early and late mitotic events in HeLa cells J Biol Chem 2003 278 51786 95 10.1074/jbc.M306275200 14523000

[B33] Hoar K Chakravarty A Rabino C Wysong D Bowman D Roy N MLN8054, a small-molecule inhibitor of Aurora A, causes spindle pole and chromosome congression defects leading to aneuploidy Mol Cell Biol 2007 27 4513 25 10.1128/MCB.02364-06 17438137 PMC1900054

[B34] Reed CA Mayhew CN McClendon AK Knudsen ES Unique impact of RB loss on hepatic proliferation: tumorigenic stresses uncover distinct pathways of cell cycle control J Biol Chem 2010 285 1089 96 10.1074/jbc.M109.043380 19887370 PMC2801236

[B35] Deshmane SL Kremlev S Amini S Sawaya BE Monocyte chemoattractant protein-1 (MCP-1): an overview J Interferon Cytokine Res 2009 29 313 26 10.1089/jir.2008.0027 19441883 PMC2755091

[B36] Finch PW Rubin JS Keratinocyte growth factor/fibroblast growth factor 7, a homeostatic factor with therapeutic potential for epithelial protection and repair Adv Cancer Res 2004 91 69 136 full_text 15327889 10.1016/S0065-230X(04)91003-2

[B37] Lademann UA Romer MU Regulation of programmed cell death by plasminogen activator inhibitor type 1 (PAI-1) Thromb Haemost 2008 100 1041 6 19132228

[B38] Baxter RC Signalling pathways involved in antiproliferative effects of IGFBP-3: a review Mol Pathol 2001 54 145 8 10.1136/mp.54.3.145 11376125 PMC1187052

[B39] Serrano M Lin AW McCurrach ME Beach D Lowe SW Oncogenic ras provokes premature cell senescence associated with accumulation of p53 and p16INK4a Cell 1997 88 593 602 10.1016/S0092-8674(00)81902-9 9054499

[B40] Pirker C Lötsch D Spiegl-Kreinecker S Jantscher F Sutterlüty H Micksche M Response of experimental malginant melanoma models to the pan-Aurora kinase inhibitor VE-465 Experimental Dermatology 2010 19 12 1040 7 10.1111/j.1600-0625.2010.01182.x 21087322

[B41] Mayer CE Haigl B Jantscher F Siegwart G Grusch M Berger W Bimodal expression of Sprouty2 during the cell cycle is mediated by phase-specific Ras/MAPK and c-Cbl activities Cell Mol Life Sci 2010 67 3299 311 10.1007/s00018-010-0379-6 20461437 PMC11115549

[B42] Sutterluty H Mayer CE Setinek U Attems J Ovtcharov S Mikula M Down-Regulation of Sprouty2 in Non-Small Cell Lung Cancer Contributes to Tumor Malignancy via Extracellular Signal-Regulated Kinase Pathway-Dependent and -Independent Mechanisms Mol Cancer Res 2007 5 509 20 10.1158/1541-7786.MCR-06-0273 17510316

[B43] Sutterluty H Chatelain E Marti A Wirbelauer C Senften M Muller U p45SKP2 promotes p27Kip1 degradation and induces S phase in quiescent cells Nat Cell Biol 1999 1 207 14 10.1038/12027 10559918

[B44] Haigl B Mayer CE Siegwart G Sutterluty H Sprouty4 levels are increased under hypoxic conditions by enhanced mRNA stability and transcription Biol Chem 2010 391 813 21 10.1515/BC.2010.082 20482313

[B45] Sutterluety H Bartl S Doetzlhofer A Khier H Wintersberger E Seiser C Growth-regulated antisense transcription of the mouse thymidine kinase gene Nucleic Acids Res 1998 26 4989 95 10.1093/nar/26.21.4989 9776764 PMC147947

